# RAE1 mediated *ZEB1* expression promotes epithelial–mesenchymal transition in breast cancer

**DOI:** 10.1038/s41598-019-39574-8

**Published:** 2019-02-27

**Authors:** Ji Hoon Oh, Ji-Yeon Lee, Sungsook Yu, Yejin Cho, Sumin Hur, Ki Taek Nam, Myoung Hee Kim

**Affiliations:** 1 Department of Anatomy, Embryology Laboratory, Yonsei University College of Medicine, Seoul, 03722 Korea; 2 Brain Korea 21 PLUS Project for Medical Science, Yonsei University College of Medicine, Seoul, 03722 Korea; 3 Severance Biomedical Science Institute and Brain Korea 21 PLUS Project for Medical Science, Yonsei University College of Medicine, Seoul, 03722 Korea

## Abstract

Breast cancer metastasis accounts for most of the deaths from breast cancer. Since epithelial-mesenchymal transition (EMT) plays an important role in promoting metastasis of cancer, many mechanisms regarding EMT have been studied. We previously showed that Ribonucleic acid export 1 (RAE1) is dysregulated in breast cancer and its overexpression leads to aggressive breast cancer phenotypes by inducing EMT. Here, we evaluated the functional capacity of RAE1 in breast cancer metastasis by using a three-dimensional (3D) culture system and xenograft models. Furthermore, to investigate the mechanisms of RAE1-driven EMT, *in vitro* studies were carried out. The induction of EMT with RAE1-overexpression was confirmed under the 3D culture system and *in vivo* system. Importantly, RAE1 mediates upregulation of an EMT marker ZEB1, by binding to the promoter region of *ZEB1*. Knockdown of ZEB1 in RAE1-overexpressing cells suppressed invasive and migratory behaviors, accompanied by an increase in epithelial and a decrease in mesenchymal markers. Taken together, these data demonstrate that RAE1 contributes to breast cancer metastasis by regulating a key EMT-inducing factor ZEB1 expression, suggesting its potential as a therapeutic target.

## Introduction

Breast cancer is one of the most commonly occurring cancers in women worldwide^1^. The main reason for death of breast cancer patients is metastasis^2^. The epithelial-mesenchymal transition (EMT), a process that is typically induced by interruption of intracellular tight junctions and loss of cell-cell contacts, is a key step in cancer metastasis^1,3,4^. During EMT, morphological changes from cobblestone-like to spindle-shaped cells are accompanied by a marked reduction in E-cadherin and increase in mesenchymal markers, such as Vimentin and N-cadherin^5–7^. Furthermore, EMT has been highlighted in breast cancer resistance to chemotherapy and/or target therapies^8–11^. Because of its importance, numerous studies have focused on these phenomena to explain and discover new mechanisms involved in breast cancer progression and metastasis; however, further studies of the regulation of EMT are required.

Ribonucleic acid export 1 (Rae1) was originally reported as a nucleocytoplasmic transport factor in yeast^12^. Since then, human *RAE1*, a homologue of the yeast Rae1^13^, was discovered as tone component of nuclear pore complexes (NPCs)^14^ and as a mitotic checkpoint regulator^15–17^. Recently, several studies demonstrated that RAE1 expression was dysregulated in breast cancer^18–20^. Furthermore, mRNA expression of RAE1 was found positively correlated to gene copy number^19^. Among genes that were amplified and overexpressed in breast cancer, several genes, such as FGFR1, IKBKB, and ERBB2, were especially considered as potential therapeutic target^21,22^. With the expectation that RAE1 would also be a useful target for cancer therapy, we carried out functional studies in breast cancer cell lines and found that RAE1 contributes to aggressive cancer cell phenotype and induces EMT^18^. Furthermore, the expression level of RAE1 was positively correlated with the histologic grading in breast cancer patients with invasive ductal carcinoma^18^. Elevated RAE1 expression indicated a poor outcome in breast cancer patients^18,20^.

In this study, we investigated how RAE1 contributes to invasion and metastasis of breast cancer, three-dimensional (3D) culture system and xenograft models. In addition, our *in vitro* studies have revealed that RAE1 induces EMT by enhancing the expression of transcription factor ZEB1. Considering that EMT enhances the metastatic potential of breast cancer, our results support the relationship between RAE1 activity and breast cancer aggressiveness.

## Results

### RAE1 overexpression enhances cell spreading in 3D culture systems and metastasis in mouse xenograft models

**Figure 1:**
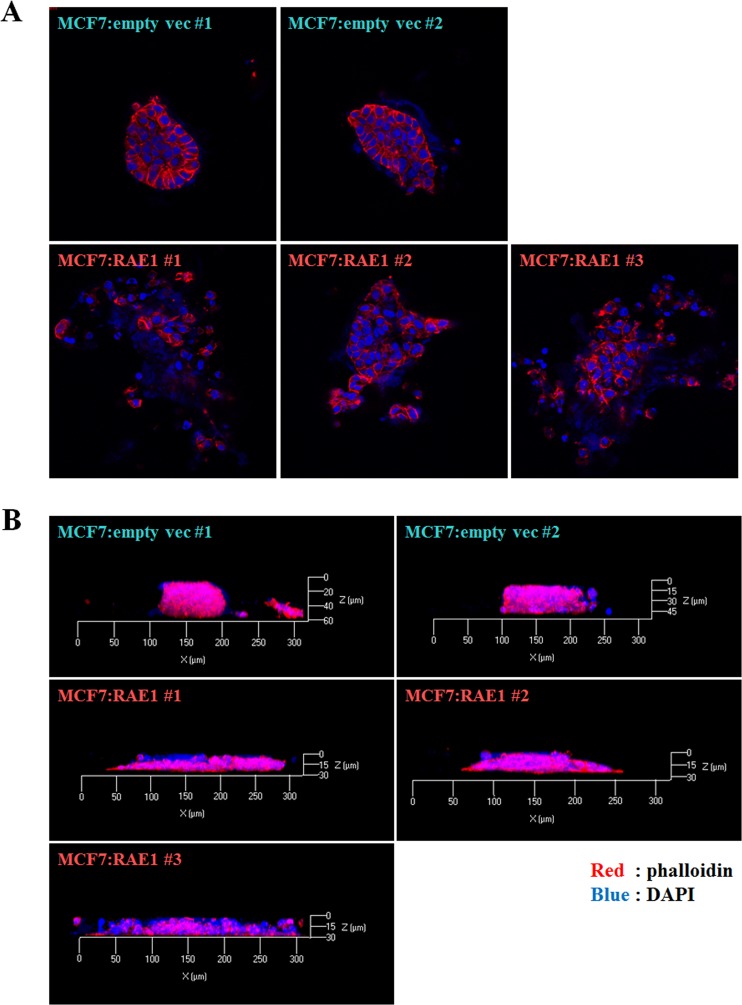
Effects of RAE1 overexpression in 3D *in vitro* culture system. (**A**,**B**) Confocal microscopy images of MCF7 cells in 3D culture system at day 10. Control (MCF7:empty vec #1 and 2) and RAE1-overexpressing MCF7 (MCF7:RAE1 #1, 2, and 3) cells were cultured in DMEM containing 4% Matrigel in a vessel coated with absolute Matrigel. Structures were stained with DAPI (blue) and phalloidin (red). The migrating features were observed in the cross-section images of control and RAE1-overexpressing MCF7 cell lines (**A**) and in the total colony structures (**B**).

To investigate the precise effects of RAE1 overexpression in breast cancer, we carried out 3D cell culture analysis with stable MCF7 cell lines overexpressing RAE1 (MCF7:RAE1 #1, 2, and 3) and empty vector (MCF7:emp vec #1, and 2). The Matrigel-embedded 3D culture system is more appropriate for structural and functional studies than the 2D culture system^23^. The results of phalloidin and DAPI staining at day 10 showed that MCF7 cells stably overexpressing RAE1 spread outwards along the extracellular matrix, whereas the control MCF7 cell lines maintained a spherical morphology without extending along the bottom line of the 3D culture vessel (Fig. 1A). In addition, confocal images representing a cross-section of the colony revealed that RAE1-overexpressing MCF7 cells were dispersed towards the outside, while control MCF7 cells gathered near the center (Fig. 1B). Serial confocal transverse section images of each stable cell line are provided in Fig. S1.

**Figure 2:**
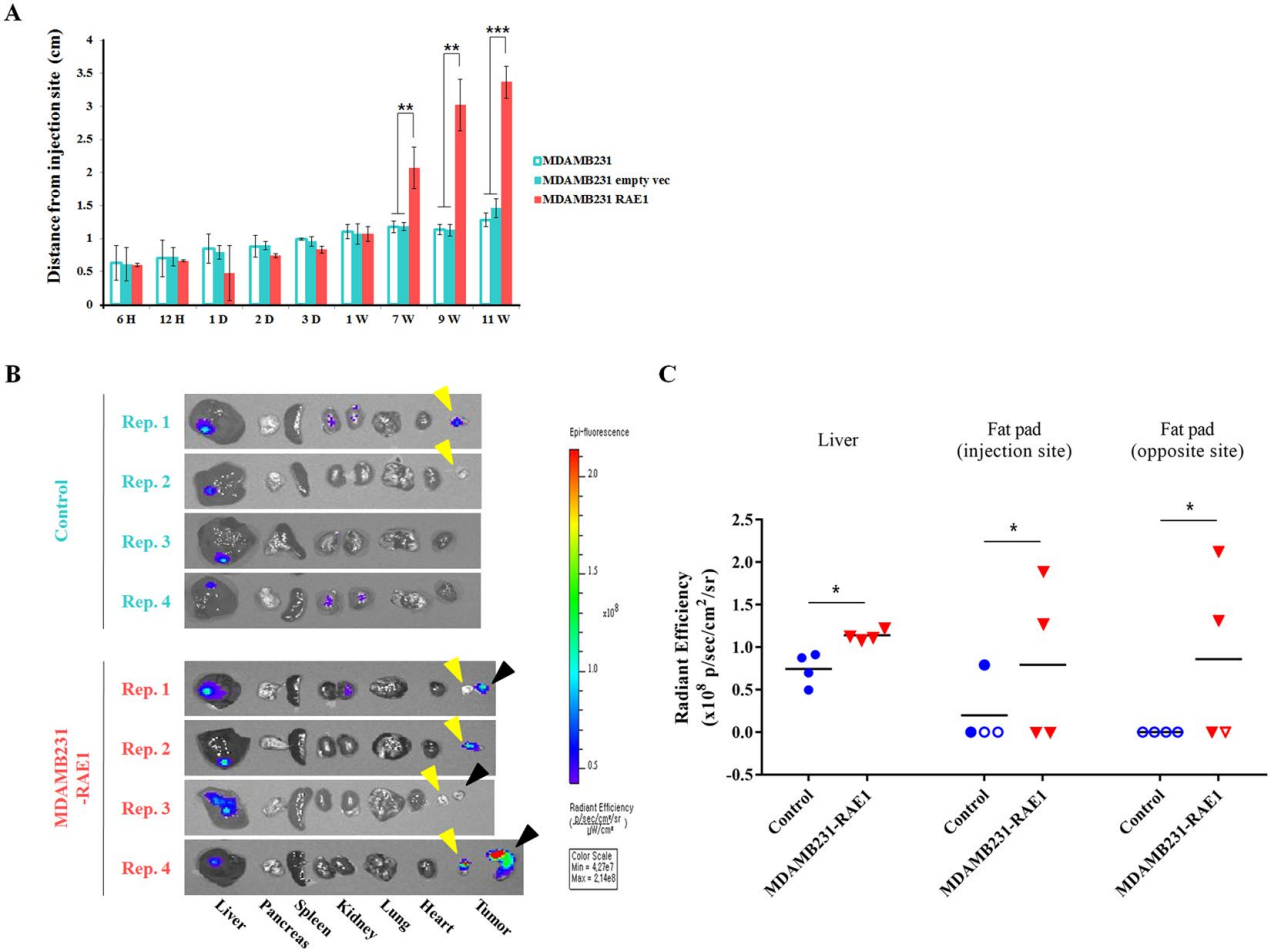
Effects of RAE1 overexpression in xenograft tumor progression. (**A**,**B**) *In vivo* xenograft models of breast cancer metastasis. Three cancer cell lines (MDA-MB-231, MDA-MB-231:empty vec, and MDA-MB-231:RAE1) were injected into the fat pads of nude mice. Four nude mice were used for each cell line. (**A**) Migration distance from 6 hrs to 11 weeks after injection. **P < 0.01, ***P < 0.001. (**B**) Measurement of metastatic spread of cancer cells to other organs (liver, pancreas, spleen, kidney, lung, and heart) at 11 weeks after injection. Yellow arrow heads indicate tumors formed at the injection site and black arrow heads indicate tumors formed at the fat pad opposite to the injection site. (**C**) Quantification analysis of signals in liver metastasis and tumors on fat pads of Fig. 1B shows average radiant efficiency. Each blue circle (injection of control cells) and red triangle (injection of RAE1 overexpressing cells) represents an individual xenograft, and empty circles and triangles indicate the no tumor has developed in the organ. In the graph, the horizontal lines represent the average value of each experimental group. *P < 0.05.

To further explore the functional role of RAE1 in breast cancer progression *in vivo*, we evaluated the effects of RAE1 on the metastasis in a breast cancer xenograft model. MDA-MB-231 cells were used in this study because of their high metastatic potential. Tumors were monitored over a period of 11 weeks, and then the distance between the injection site and final position was measured as shown in Fig. 2A. Short-term xenograft (6 hrs–1 week) did not show significant migration, while long-term xenograft (7–11 weeks) showed significant migratory abilities by RAE1-overexpressing cells. The investigation of tumor cell spreading from the primary tumor cells to other sites at 11 weeks showed that xenograft mice injected with control cells formed primary tumors at the injection site (2 out of 4 mice) but did not form metastatic tumors at any other organs throughout the body except the liver (Fig. 2B,C). On the other hand, xenograft mice with RAE1-overexpressing cells formed primary tumors at the injection site (4 out of 4 mice) and metastatic tumors at the fat pad opposite to the injection site (3 out of 4 mice) (Fig. 2B,C). Although the metastatic tumors were not found during this period of time in distal organs such as kidney and lung in both groups, all mice injected with RAE1-overexpressing cells showed a stronger signal for liver metastases (Fig. 2C). Together, these results support that RAE1 accelerates tumor metastasis *in vivo*.

### Upregulation of RAE1 enhances the expression of *ZEB1* by binding to the promoter region

**Figure 3:**
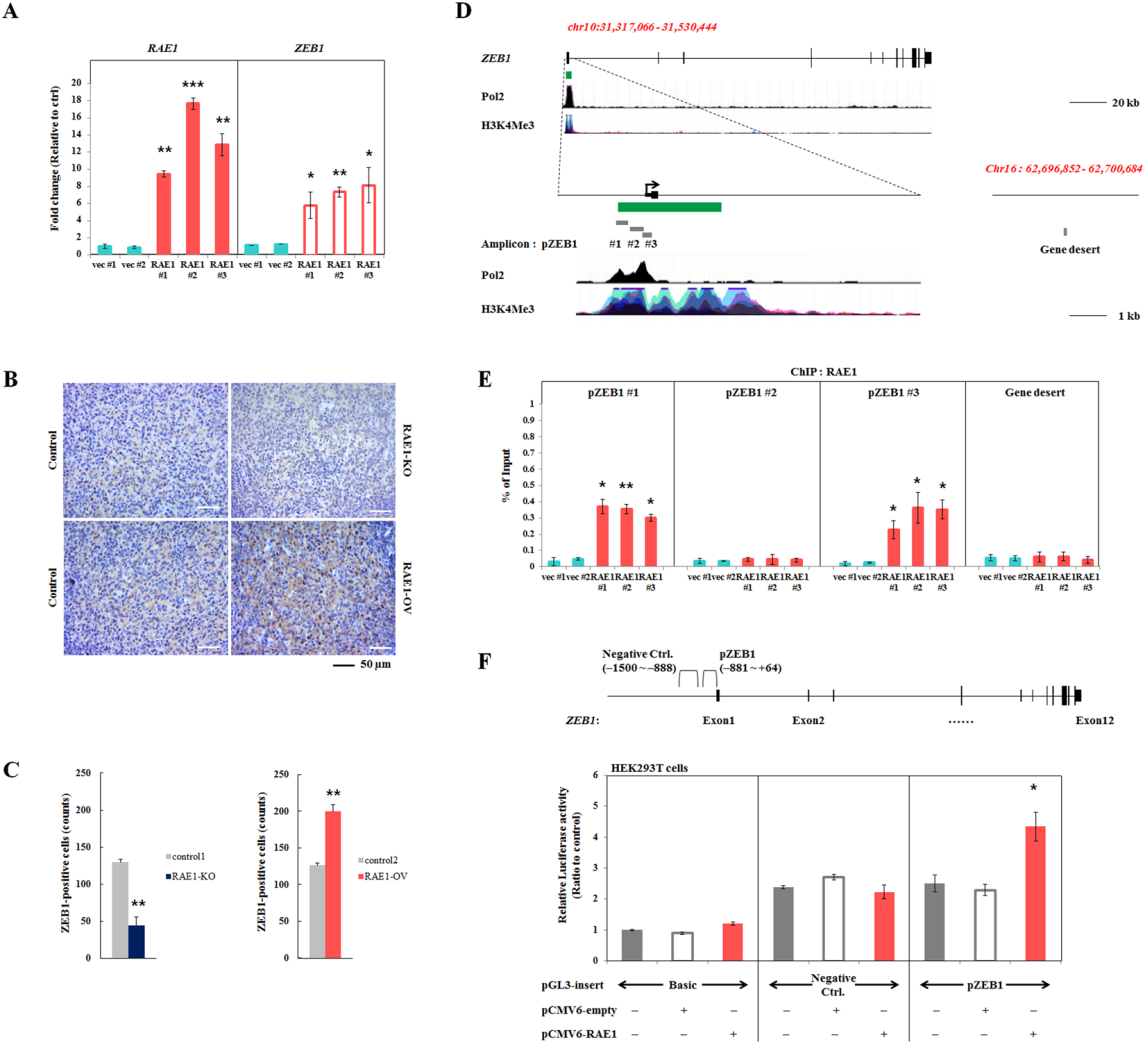
Positive correlation of RAE1 and ZEB1 *in vitro* and *in vivo*. (**A**) *RAE1* and *ZEB1* mRNA expression levels in RAE1-overexpressing MCF7 cells and control cells. (**B**) Immunohistochemistry of tumor tissues from xenograft-bearing mice with RAE1-manupulation to display the distribution of ZEB1 in the tumor sections using anti-ZEB1 antibody. (**C**) Quantification of the ZEB1-positive cells was performed. ZEB1-positive cells were measured in 10 frames each experiment. (**D**) Map of the *ZEB1* promoter region and gene desert. Green bar indicates the CpG islands and gray box shows ChIP amplicons (pZEB1 #1: −881 to −574, #2: −537 to −165 and #3: −164 to +64). H3K4Me3 and Pol2 signals were derived from ENCODE (https://genome.ucsc.edu). (**E**) Quantitative interpretation of ChIP-qPCR data. Chromatin was extracted from MCF7 cells stably overexpressing RAE1 and control cells. ChIP products were used in qPCR for pZEB1 #1, #2, and #3. An amplicon for a gene desert was included as a negative control. Data are shown as % of input, after normalization with IgG. (**F**) Luciferase activity of control and *ZEB1* promoter construct, with and without transfected RAE1 plasmid in HEK293T cells. Relative luciferase units (RLU) were measured and normalized against internal control (Renilla) luciferase activity. All experiments were performed in triplicate. *P < 0.05, **P < 0.01, ***P < 0.001.

To investigate the molecular mechanisms underlying the role of RAE1 in mediating cancer metastasis, we performed gain of function studies using *in vitro* models. Among various breast cancer cell lines, we found that RAE1 is expressed highly in BT474, but it is expressed relatively low in MDA-MB-453, T47D, and MDA-MB-231 (Fig. S2A,B). We confirmed the subcellular localization of endogenous and exogenous RAE1 in several different cell lines (Fig. S2C,D) and concluded that forced expression of RAE1 does not lead to mislocalization of abnormal protein product. Recent studies on the NPC components and their association with gene expression regulation suggest that high concentration of RAE1 at the peripheral portion of the nucleus may play a role as a transcription regulator^24–26^. As RAE1 has been shown to induce EMT signals and promote invasion and migration abilities, we determined the expression levels of several EMT-associated transcription factors (Fig. S3) and found that *ZEB1* mRNA levels were significantly upregulated by RAE1 overexpression (Fig. 3A). Furthermore, in order to confirm the positive correlation between RAE1 and ZEB1 in an *in vivo* system, IHC was performed with anti-ZEB1 antibody in tumor tissues retrieved from the xenograft experiment. In the MDA-MB-231 xenograft tumor tissues, ZEB1 was expressed mainly in the nucleus. The number of ZEB1-positive cells decreased from 129.5 ± 4.42 to 44.6 ± 11.45 in RAE1-knockdowned tumors, but increased from 126.3 ± 2.80 to 199.6 ± 9.03 in RAE1-overexpressing tumors. This may be an indirect evidence for the altered expression of ZEB1 through RAE1 regulation (Fig. 3B,C).

To determine whether RAE1 may regulate for *ZEB1* expression, the ability of RAE1 to bind the *ZEB1* promoter was determined by ChIP assay. PCR amplicon sites were designed near the putative promoter region of *ZEB1* (Fig. 3D). ChIP-qPCR data showed that overexpression of RAE1 led to increased binding of RAE1 in the −880 to −157 bp and −164 to +64 bp amplicon sites (pZEB1 #1 and #3) (Fig. 3E). To further delineate the effects of RAE1 on *ZEB1* transcriptional activity, we performed dual luciferase assay by cloning *ZEB1* promoter region (from −881 up to + 64 bp downstream of the TSS) and negative control (from −1500 up to −888 bp downstream of the TSS) into the luciferase vector. The *ZEB1* promoter activity was increased by overexpression of RAE1 compared to negative control in HEK293T cells (Fig. 3F). Collectively, these results suggest RAE1 positively regulates *ZEB1* expression during cancer progression.

### ZEB1 is a mediator for RAE1-induced EMT, invasion and migration in breast cancer

**Figure 4:**
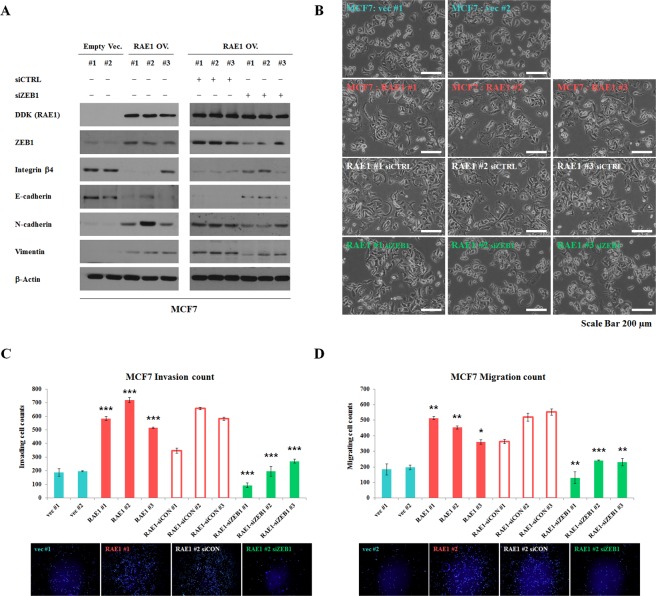
ZEB1 mediates RAE1-induced EMT and invasion/migration abilities. (**A**) Western blotting analysis for epithelial and mesenchymal markers in stable RAE1-overexpressing MCF7 cells, with treatment of siCTRL or siZEB1. Full-length blots are presented in Fig. S4. (**B**) Effect of ZEB1 knockdown on cell morphological changes. For these experiments, 3.5 × 10^5^ cells were seeded onto 6-well plates, transfected with siZEB1 or siCTRL for 48 hrs, and then imaged using a microscope for any morphological changes. Scale bar = 200 μm. (**C**,**D**) Matrigel invasion and migration assay in stable RAE1-overexpressing MCF7 cells, with treatment of siCTRL or siZEB1. For these experiments, 5 × 10^4^ cells were placed in each chamber. After incubation for 72 hrs, invading or migrating cells were stained with DAPI and analyzed via fluorescent microscopy. *P < 0.05, **P < 0.01, ***P < 0.001.

In a previous study, we have shown that overexpression of RAE1 in epithelial-like MCF7 and T47D induces EMT-like morphological changes and EMT marker expression^18^. In an opposite way, knockdown of RAE1 in mesenchymal-like MDA-MB-231 cells reduced cancer cell invasion and migration^18^. Here, to examine whether RAE1-induced metastatic capability was mediated by ZEB1, siRNA was used to silence *ZEB1* gene expression. We have previously shown that overexpression of RAE1 in MCF7 cells also altered EMT-related marker levels and showed more distinct spindle-shape morphology (Fig. 4A, left 6 lanes; Fig. 4B, rows 1 and 2). Knockdown of *ZEB1* in RAE1-overexpressing MCF7 cells promoted a reversal of EMT by increasing the expression of epithelial markers (Integrin β4 and E-cadherin) and decreasing the levels of mesenchymal markers (N-cadherin and Vimentin) (Fig. 4A, right 6 lanes; Fig. 4B, rows 3 and 4). In addition, silencing ZEB1 repressed RAE1-driven invasion and migration abilities (Fig. 4C,D). The effect of ZEB1 knockdown on the morphological and molecular changes was similarly observed in RAE1-overexpressing T47D and MDA-MB-231cells (Figs S5 and S6). Together, these data suggest that ZEB1 functions as a key component of RAE1-mediated EMT in breast cancer.

## Discussion

In this study, we evaluated the metastatic properties of RAE1-overexpressing breast cancer cells both in 3D culture systems and *in vivo* xenograft models, and further demonstrated the molecular mechanisms underlying RAE1-mediated tumor progression. Our data revealing RAE1-mediated ZEB1-transcription regulation suggest that ZEB1, an EMT inducing transcription activator, contributes to the development of RAE1-driven EMT.

RAE1 is a component of the NPC. NPCs are large multi-protein structures that are present in the double membrane of the nuclear envelope mediating trafficking between the nucleoplasm and cytoplasm^27–29^. Alterations in nucleoporins, which compose each NPC, are frequently associated with particular defects in development and disease, and the resulting phenotypes are typically thought to be consequences of disturbed activity of nuclear transport^24,29^. Particularly, primary human specimens derived from different forms of cancer revealed dysregulation of mRNA export. Various components of mRNA export and its related factors contribute to the preferential export of transcripts encoding proteins involved in proliferation, survival, metastases, and invasion^30^.

Alternatively, NPCs can regulate the transcription levels of particular genes in a transport-independent manner^24,26^. Several studies have suggested that interactions between genes and the NPC modulate both the definition of hetero- and euchromatin boundaries and transcription^25^. Thus, NPCs are essential for controlling not only transport between the nucleoplasm and cytoplasm, but also organization of the genome and specific gene transcription^31^. The potential function of RAE1 as a transcription activator has not been reported before. However, some previous studies demonstrated the role of nuclear pore proteins (Nups), which interact with RAE1, as potential regulator of gene transcription. For example, Nup210 positions its target genes at the nuclear periphery, an environment where gene transcription can be controlled^32,33^. Here, we provide evidence that RAE1 regulates gene transcription by binding to the promoter region of a particular gene, *ZEB1*. Although we have realized the analysis of RAE1 expression in the Cancer Cell Line Encyclopedia (CCLE) databank did not find any correlation between *RAE1* and *ZEB1* mRNA expression nor with the breast cancer subtypes (Fig. S7), it may seem quite plausible to say that the regulation of *ZEB1* expression by RAE1 is cell context-specific and/or may depend on the threshold of RAE1 expression. Taking this into account, our data showing the possibility of modulating *ZEB1* expression by RAE1 suggest that the association of these two molecules may be important under certain conditions.

Apart from its function as a transcription factor, it is possible that RAE1 interacts with cytoplasmic components such as the cytoskeleton and contributes to genome organization and gene expression. RAE1, as a nuclear exporter, is expected to only be present in nuclear pores. However, according to public available databases (e.g. The Human Protein Atlas), RAE1 is present in the nucleoli fibrillar center and in the nucleus (Fig. S8). In addition, our ICC results show that RAE1 can be detected in the cytoplasm. (Fig. S2). The cytoskeleton is a well-organized dynamic cellular architecture that is typically involved in signal transduction in the cytoplasm^34^. In this regard, NPCs can act as docking sites for chromatin, ultimately contributing to the organization of the global topology of chromosomes in close association with other elements of the nuclear envelope^25^.

Based on the facts that the dysregulation of many NPC components is found in the different types of cancer and that various nuclear factors interacting with them, such as chromosomal maintenance 1 (CRM1, also known as exportin1), karyopherin family (KPNA2 and KPNB1), and chromosome segregation gene, contribute to the progression of cancer^29,35–37^, more attention is being focused on developing therapeutic drugs using transport factors^35,38^. In particular, CRM1/XPO1 is proposed as a promising drug candidate^39,40^, and is likely to bind to RAE1, either directly or indirectly (Fig. S9). Therefore, understanding the RAE1 function and mechanisms of action will be valuable enough to consider the value of therapeutic use. In conclusion, our study demonstrated that RAE1 promotes progression of breast cancer cells by activating *ZEB1* at the transcription level and revealed the positive correlation between RAE1 and ZEB1 in breast cancer metastasis.

## Methods

### Cell lines

MCF7 and MDA-MB-231 breast cancer cell lines stably overexpressing RAE1 were generated and cultured as previously described^18^. For ZEB1 knockdown experiment, transient siRNA-mediated knockdown was carried out with HiPerFect transfection reagent (Qiagen, Hilden, Germany) according to the manufacturer’s protocol for 48 hrs. ZEB1 siRNA (Genolution, Seoul, Korea) or control siRNA were used to a final concentration of 40 nM.

### Three-dimensional (3D) *in vitro* cell culture

To analyze cellular growth in 3D culture, an eight-well chamber slide was pre-coated with 50 μL of Matrigel^TM^ (BD, San Jose, CA, USA) and incubated at 37 °C for 30 min to allow for gel formation. While the Matrigel was solidifying, 5 × 10^3^ cells were diluted in cell culture medium (final concentration: 2.5 × 10^4^ cells/mL) and mixed with 4% Matrigel-containing medium in a 1:1 ratio. The cell mixture was placed on top of the solidified Matrigel. Medium containing 2% Matrigel was changed every 3–4 days. Confocal images were acquired on the 10th day of culture after staining with anti-phalloidin (Invitrogen, Carlsbad, CA, USA) and fluorochrome 4′,6-diamidino-2-phenylindole (DAPI). Images were captured with a 40 × C-Apochromat water immersion lens on a Zeiss LSM 700 Confocal, using Zen 2011 software (Carl Zeiss, Oberkochen, Germany). For 3D images, z-stack scans were collected by incremental stepping through the 3D sample using a focal drive. The step size was automatically calculated with Zen 2011 software.

### Tumor xenograft experiment

For xenograft experiments, male BALB/c nude mice were purchased from The Orient Bio, Inc. (Sungnam, Gyungki–do, Korea). RAE1-overexpressing MDA-MB-231 cells, and the same amount of MDA-MB-231 stable cells overexpressing empty vector and parent MDA-MB-231 cells, were used. For each mouse, 5 × 10^5^ cells were stained with XenoLight Dir Fluorescence dye (PerkinElmer, Waltham, MA, USA), washed twice with cold sterile PBS without calcium chloride and magnesium chloride (Sigma-Aldrich, St. Louis, MO, USA), and mixed 50:50 (v/v) with Matrigel. The cells were injected into the right fat pad of 7 week-old male BALB/c nude mice to establish primary tumors. Each mouse was analyzed using IVIS (Caliper Life Sciences, Hopkinton, MA, USA) at 710 nm for excitation and 760 nm for emission from 6 hrs after injection up to 11 weeks. Mice were sacrificed in a CO_2_ chamber at 11 weeks after injection, and the liver, pancreas, spleen, kidney, lung, and heart tissues were collected to analyze metastasis. All animal procedures were approved by the Institutional Animal Care Committee at Yonsei University, and were carried out in accordance with the guidelines and regulations set by the ethics committee. Animals were then euthanized and selected tissues were processed for histology.

### Immunohistochemistry (IHC) analysis

IHC analyses were performed as previously described with minor modification^41^. Briefly, slides were deparaffinized and rehydrated through series of graded ethanol. Antigen retrieval was performed using a pressure cooker. Endogenous peroxidase activity was blocked by incubation with 3% H_2_O_2_ for 30 min then incubated with protein blocking solution (Dako, Glostrup, Denmark) for 1 hour at room temperature. Primary antibody was incubated in a humid chamber at 4 °C overnight, and then slides were incubated with secondary rabbit IgG (Dako) for 15 min at room temperature, and developed with Dako Envision + System-HRP DAB (Dako). Anti-ZEB1 (Abcam, Cambridge, UK; dilution ratio 1:2000) was purchased. After counterstaining with Meyer’s Hematoxylin (Sigma), slides were mounted with mounting solution (Electron Microscopy Sciences, Hatfield, PA, USA). Quantitation of ZEB1-positive cells was done and then statistical analyses were performed with JMP software. Student’s *t*-tests were used to analyze differences between means. Data are represented as mean ± SEM.

### Immunocytochemistry (ICC) analysis

ICC analyses were performed, with Abcam’s ICC protocol. An anti-RAE1 antibody (Abcam) was used to detect RAE1 protein. The nucleus was counterstained with DAPI. Images were captured with a 40 × C-Apochromat water immersion lens on a Zeiss LSM 700 Confocal, using Zen 2011 software.

### Real-time PCR

Real-time PCR analysis was performed as previously described^42^. For quantitative PCR analysis, the StepOnePlus^TM^ Real-Time PCR System (Applied Biosystems, Foster City, CA, USA) and Power SYBR Green PCR Master Mix (Applied Biosystems) kits were used. All samples were run in triplicate, and *RAE1* and *ZEB1* expression levels were normalized relative to that of *β-Actin*, which was used as an internal loading control. Primers for PCR are listed in Table 1.

**Table 1:** Primer sequences used for real-time PCR.

Genes	Sequence (5′ → 3′)
*RAE1*	F- CAA CCT CAG GTT TTG GAA CC
	R- CGA TGC CGT AAA CAC TTT GC
*ZEB1*	F- TCC TCT CGA ATG AGC ACG
	R- CTT GCT CAC TAC TCT CG
*β-Actin*	F- CATGTTTGAGACCTTCAACACCCC
	R- GCCATCTCCTGCTCGAAGTCTAG

### Western blotting and antibodies

Western blot analyses were performed as previously described^18^. Anti-RAE1 (Abcam), anti-DDK-tag mouse monoclonal antibody (Origene, Rockville, MD, USA), anti-ZEB1 (Abcam), anti-E-cadherin (Abcam), anti-Integrinβ4 (Abcam), anti-β-catenin (BD, Franklin Lakes, USA), anti-N-cadherin (Abcam), anti-Vimentin (Sigma, St. Louis, MO, USA), and anti-β-Actin (Sigma) antibodies were used to detect each protein.

### Matrigel invasion and migration assays

The Matrigel^TM^ (BD) invasion and migration assays were performed previously described^18^.

### Chromatin immunoprecipitation (ChIP) assay

ChIP analysis was performed as previously described^42^ with minor modifications. Chromatin was prepared from stable RAE1-overexpressing and control breast cancer cell lines. Briefly, 1 × 10^6^ cells were cross-linked with 1% formaldehyde for 15 min, followed by the addition of glycine at 125 mM. Chromatin was sheared by sonication to fragments averaging between 0.5 and 1 kb in buffer containing 1% SDS, 1% Triton X-100, 0.1% sodium deoxycholate, 10 mM EDTA, 50 mM Tris-HCl (pH 8.0), and protease inhibitor cocktail (Roche Applied Science, Basel, Switzerland). Chromatin was pre-cleared with protein A/G beads containing 50% slurry (Santa Cruz Biotechnology, Dallas, TX, USA) and salmon sperm DNA, followed by immunoprecipitation with anti-RAE1 antibody (Abcam) coupled to protein A/G beads under each experimental condition. Nonimmune mouse IgG (Santa Cruz Biotechnology) was used as a control. ChIP-PCR data are shown as the percentage of input after normalization with IgG. Primers for ChIP-PCR are listed in Table 2.

**Table 2:** Primer sequences used for ChIP-PCR assay.

Amplicon sites	Sequence (5′ → 3′)
pZEB1 #1	F- GGA TCC CAC GGT TCT ACG C
	R- GCG ACC GGA GAG AGG CTA
pZEB1 #2	F- CTC ATC AAG GGA ACT CCC CG
	R- GAA TTG AGG GGC GAG GGA AA
pZEB1 #3	F- CCC ACC ACA CCT GAG GAA AA
	R- CAT GAT CCT CTC GCT TGT GTC
Gene desert	F- TGG TGG TCT GCC TTC TGC CAG T
	R- TCA CGT GGG AGG AAG AAG TAG GGC

### Dual luciferase assay

Dual luciferase assay was performed as described previously^43^. Genomic DNA fragment of the *ZEB1* promoter region was cloned into the pGL3-Basic vector (Promega, Madison, WI, USA) using KpnI and HindIII sites. Control pGL3-Basic vector or the pGL3-ZEB1 constructs were transfected into HEK293T cells with the Renilla luciferase vector.

### *In silico* analysis

The STRING web-accessible database version 10.5 (https://string-db.org) was used to evaluate RAE1 interaction partners in various human tissues. The Human Protein Atlas (https://www.proteinatlas.org) was used to determine the localization of RAE1 in several cell lines.

### Statistical analysis

Data are expressed as the mean values with the standard error of the mean. Statistical differences were determined by Student’s *t*-test. A *P*-value of < 0.05 was considered statistically significant.

## Supplementary information

Supplementary Information
