# Supplementary material for: RAE1 mediated ZEB1 expression promotes epithelial–mesenchymal transition in breast cancer
Source: Sci Rep. 2019 Feb 27;9:2977. doi: 10.1038/s41598-019-39574-8 (PMC6393568; doi:10.1038/s41598-019-39574-8)
Supplement: Supplementary file 1 — Supplementary Information [file 41598_2019_39574_MOESM1_ESM.pdf]

Supplementary Information

RAE1 mediated *ZEB1* expression promotes epithelial–mesenchymal transition in breast cancer

Ji Hoon Oh[1,2], Ji-Yeon Lee[1], Sungsook Yu[3], Yejin Cho[3], Sumin Hur[3], Ki Taek Nam[3*] and Myoung Hee Kim[1,2*]

Supporting information

Figure S1. Serial confocal section images of RAE1-overexpressing MCF7 cells. (A and B) Control (MCF7:empty vec #1 and 2) and (C−E) RAE1-overexpressing MCF7 (MCF7:RAE1 #1, 2, and 3) cells were cultured in DMEM containing 4% Matrigel in a vessel coated with absolute Matrigel. Structures were stained with DAPI (blue) and phalloidin (red).

Figure S2. Expression level and subcellular localization of RAE1 in various breast cancer cell lines. (A) Gene expression of RAE1 in breast cancer cell lines from the Cancer Cell Line Encyclopedia (CCLE, https://portals.broadinstitute.org/ccle). Among various cell lines, *RAE1* expression was the highest in BT474 (RNAseq: 7.264051) and lower in order of MCF7 (RNAseq: 5.742407), T47D (RNAseq: 4.745019), and MDA-MB-231 (RNAseq: 4.341379). Each circle represents an individual cell line. (B) A quantitative qPCR for *RAE1* in different breast cancer cell lines (MCF7, T47D, BT474, MDA-MB-231, MDA-MB-453, SKBR3, HCC1428) and MCF10A non-tumorigenic epithelial cell line. The bar indicates the ratio of increased expression in each breast cancer cell line compared with normal breast cell line (MCF10A). * $P < 0.05$, ** $P < 0.01$. (C) Localization of RAE1 in MDA-MB-231 cells stably overexpressing RAE1 (MDA-MB-231:RAE1 #1, #2, and #3) compared to controls (MDA-MB-231:empty vec #1 and #2). When RAE1 was overexpressed in MDA-MB-231 cells, the nuclear distribution of RAE1 spread out into the cytoplasm. Fluorescent microscopy images show protein levels of RAE1 (red), DAPI (blue), and merge (violet). Scale bar = 20 µm. (D) ICC analysis show subcellular localization of RAE1 (red signals) in MCF7, MDA-MB-231, and BT474. These data show that RAE1 is mainly detected in the nucleus in cell lines with a low endogenous expression level of RAE1, such as MDA-MB-231 or MCF7, whereas RAE1

is found both in nucleus and cytoplasm in BT474 where endogenous expression of RAE1 is high. Blue signals indicate DAPI (nuclear). Scale bar = 100 μm.

Figure S3. Expression level of genes associated with cancer progression in RAE1-overexpressing MCF7 and MDA-MB-231 cell lines. The isolated RNA from the RAE1-overexpressing MCF7 (A), MDA-MB-231 (B) and control cell lines were used for analysis. Transcription levels of several genes associated with cancer progression were determined by RT-PCR using indicated primers.

Figure S4. Full-length western blot results using stable RAE1-overexpressing MCF7 cells with siCTRL or siZEB1 treatment are shown. First, the overexpression of RAE1 and knockdown of ZEB1 was confirmed with corresponding antibodies, and then Integrin β4, E-cadherin, N-cadherin, and Vimentin antibodies were used for EMT analysis. β-Actin was used as control. The sizes of EMT markers were very similar, so it was inevitable to run the samples on different blots. Furthermore, 50 μg of cell lysate was used for the detection of protein levels of RAE1, ZEB1, Integrin β4, E-cadherin, N-cadherin, and Vimentin, whereas only 10 μg of cell lysate was used for β-Actin.

Figure S5. ZEB1 mediates RAE1-induced EMT and invasion/migration abilities in T47D cells. (A) Western blotting analysis for epithelial and mesenchymal markers in stable RAE1-overexpressing T47D cells, with treatment of siCTRL or siZEB1. (B) Effect of ZEB1 knockdown on cell morphological changes. For these experiments, 3.5×10^5 cells were seeded onto 6-well plates, transfected with siZEB1 or siCTRL for 48 hrs, and then imaged using a microscope for any morphological changes. Scale bar = 200 μm. (C and D) Matrigel invasion and migration assay in stable RAE1-overexpressing T47D cells, with treatment of

siCTRL or siZEB1. For these experiments, 5×10^4 cells were placed in each chamber. After incubation for 72 hrs, invading or migrating cells were stained with DAPI and analyzed via fluorescent microscopy. * $P < 0.05$, ** $P < 0.01$, ***$P < 0.001$.

Figure S6. ZEB1 mediates RAE1-induced EMT and invasion/migration abilities in MDA-MB-231 cells. (A) Western blotting analysis for epithelial and mesenchymal markers in stable RAE1-overexpressing MDA-MB-231 cells, with treatment of siCTRL or siZEB1. (B) Effect of ZEB1 knockdown on cell morphological changes. For these experiments, 3.5×10^5 cells were seeded onto 6-well plates, transfected with siZEB1 or siCTRL for 48 hrs, and then imaged using a microscope for any morphological changes. Scale bar = 200 μm. (C and D) Matrigel invasion and migration assay in stable RAE1-overexpressing MDA-MB-231 cells, with treatment of siCTRL or siZEB1. For these experiments, 5×10^4 cells were placed in each chamber. After incubation for 6–12 hrs, invading or migrating cells were stained with DAPI and analyzed via fluorescent microscopy. * $P < 0.05$, ** $P < 0.01$, ***$P < 0.001$.

Figure S7. Pearson correlation between *RAE1* and *ZEB1* expression in various breast cancer cell lines. There was no any correlation between *RAE1* and *ZEB1* expression in (A) triple-negative and (B) LumB/HER2 subtypes of breast cancer cell lines from the Cancer Cell Line Encyclopedia (CCLE, https://portals.broadinstitute.org/ccle). Each circle represents an individual cell line.

Figure S8. Localization of RAE1 in various cell lines. RAE1 localization (green signals) was analyzed using *in silico* data retrieved from a web-accessible database (The Human Protein Atlas; https://proteinatlas.org). Various expression positions (nucleoli, nucleus, and

cytoplasm) were confirmed in several cell lines (CACO-2;colon, PC-3;prostate, and U-2 OS;bone). Blue signals indicate DAPI (nuclear) and red signals represent microtubules.

Figure S9. Interaction partners of RAE1 and XPO1. RAE1 and XPO1 interaction components were analyzed using *in silico* data retrieved from a web-accessible database (STRING version 10.5; https://string-db.org), various RAE1 interaction partners were confirmed, many of which were nuclear pore proteins, and were found to also interact with CRM1.

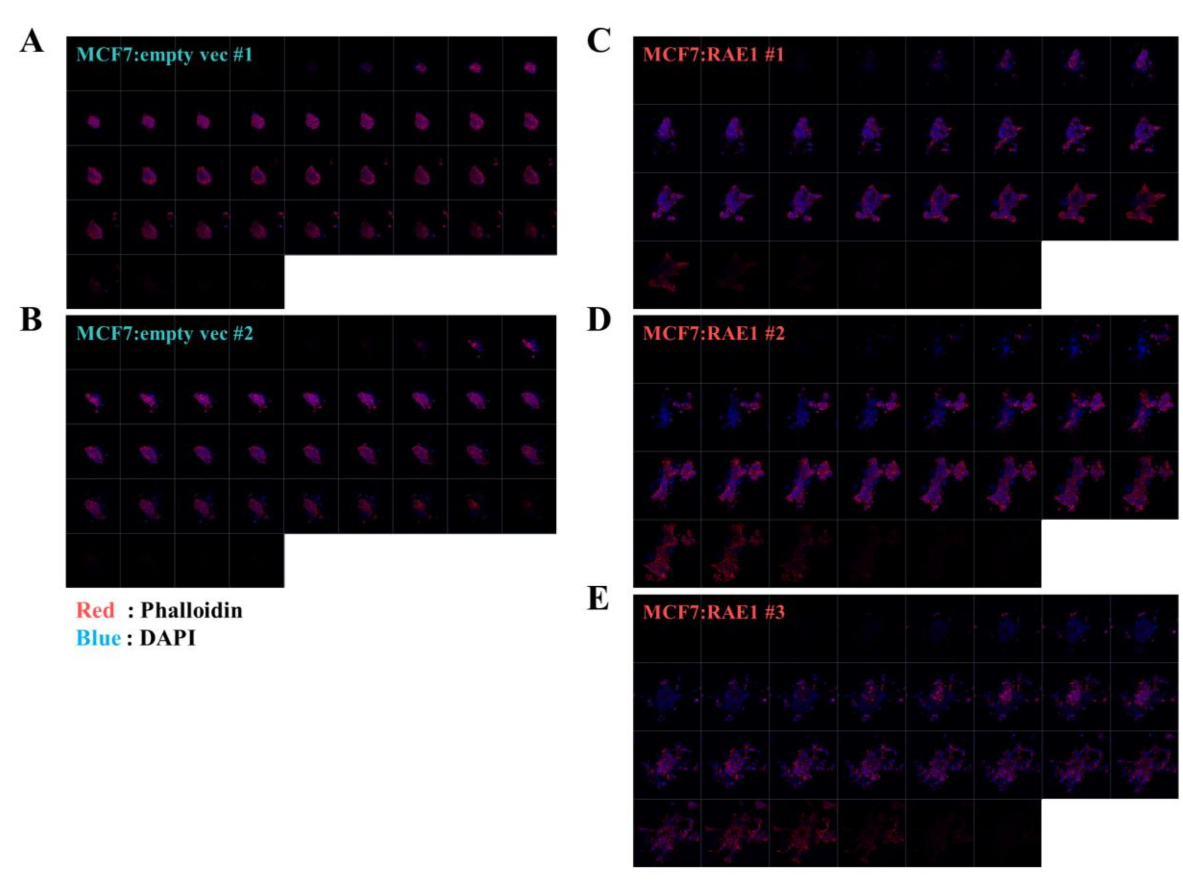
A
MCF7:empty vec #1
B
MCF7:empty vec #2
Red : Phalloidin
Blue : DAPI
C
MCF7:RAE1 #1
D
MCF7:RAE1 #2
E
MCF7:RAE1 #3

Figure S1.

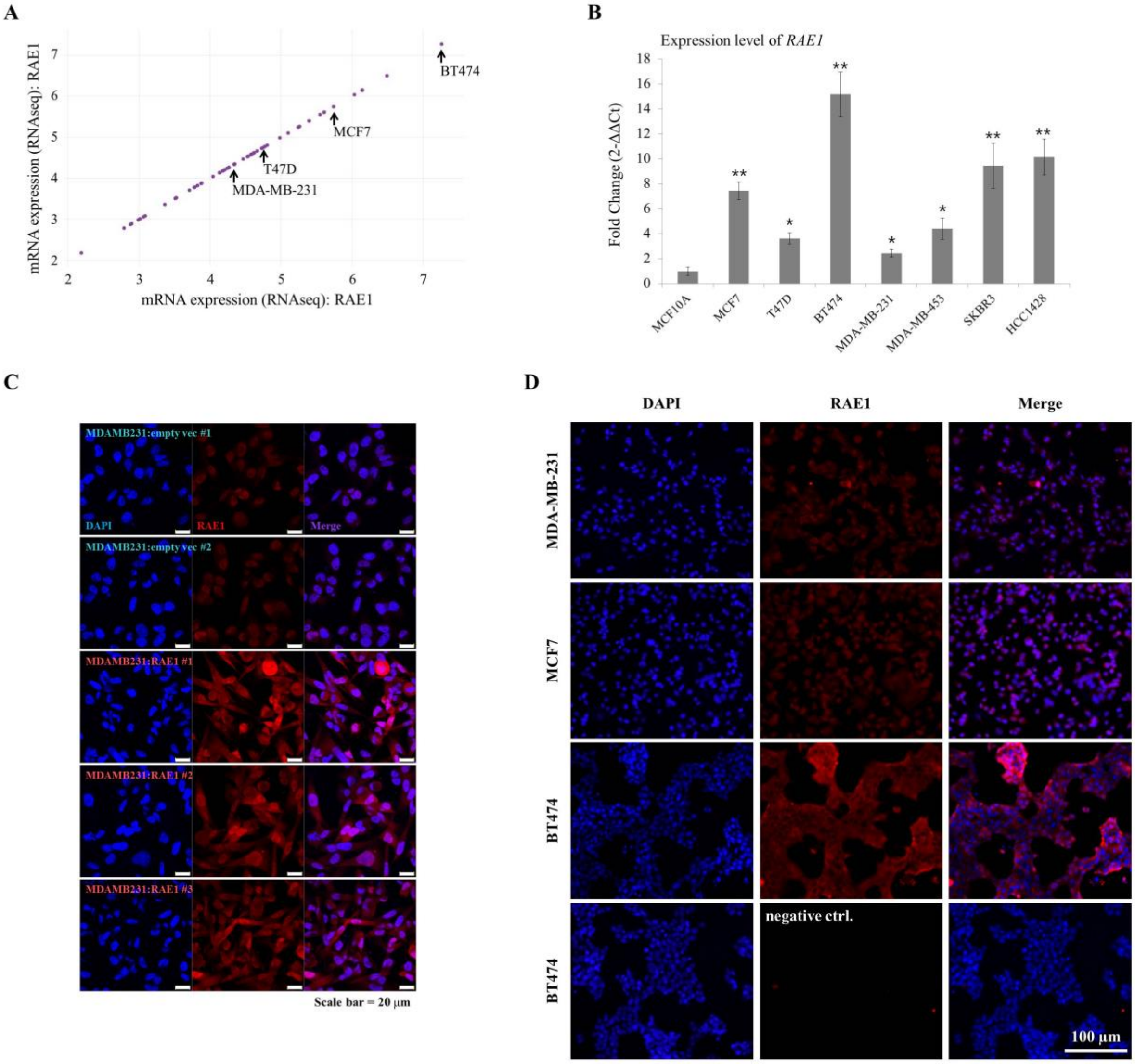
A
mRNA expression (RNAseq): RAE1
mRNA expression (RNAseq): RAE1
BT474
MCF7
T47D
MDA-MB-231
B
Expression level of RAE1
Fold Change (2-ΔΔCt)
MCF10A
MCF7
T47D
BT474
MDA-MB-231
MDA-MB-453
SKBR3
HCC1428
C
MDAMB231:empty vec #1
DAPI
RAE1
Merge
MDAMB231:empty vec #2
MDAMB231:RAE1 #1
MDAMB231:RAE1 #2
MDAMB231:RAE1 #3
Scale bar = 20 μm
D
DAPI
RAE1
Merge
MDA-MB-231
MCF7
BT474
BT474
negative ctrl.
100 μm

Figure S2.

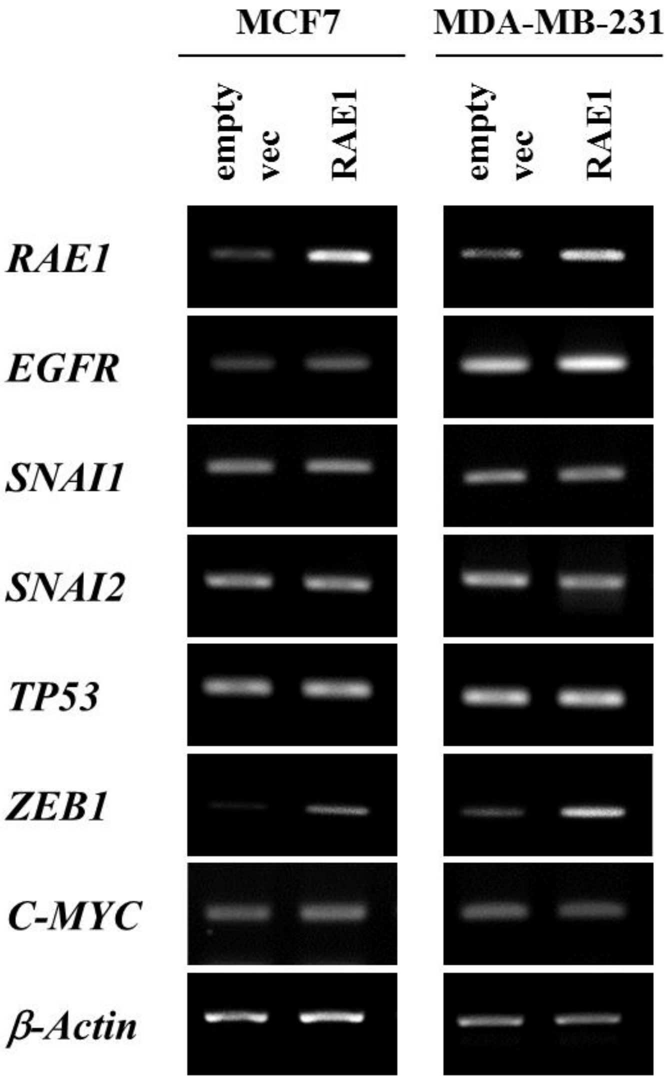
MCF7
MDA-MB-231
empty vec
RAE1
empty vec
RAE1
RAE1
EGFR
SNAI1
SNAI2
TP53
ZEB1
C-MYC
β-Actin

Figure S3.

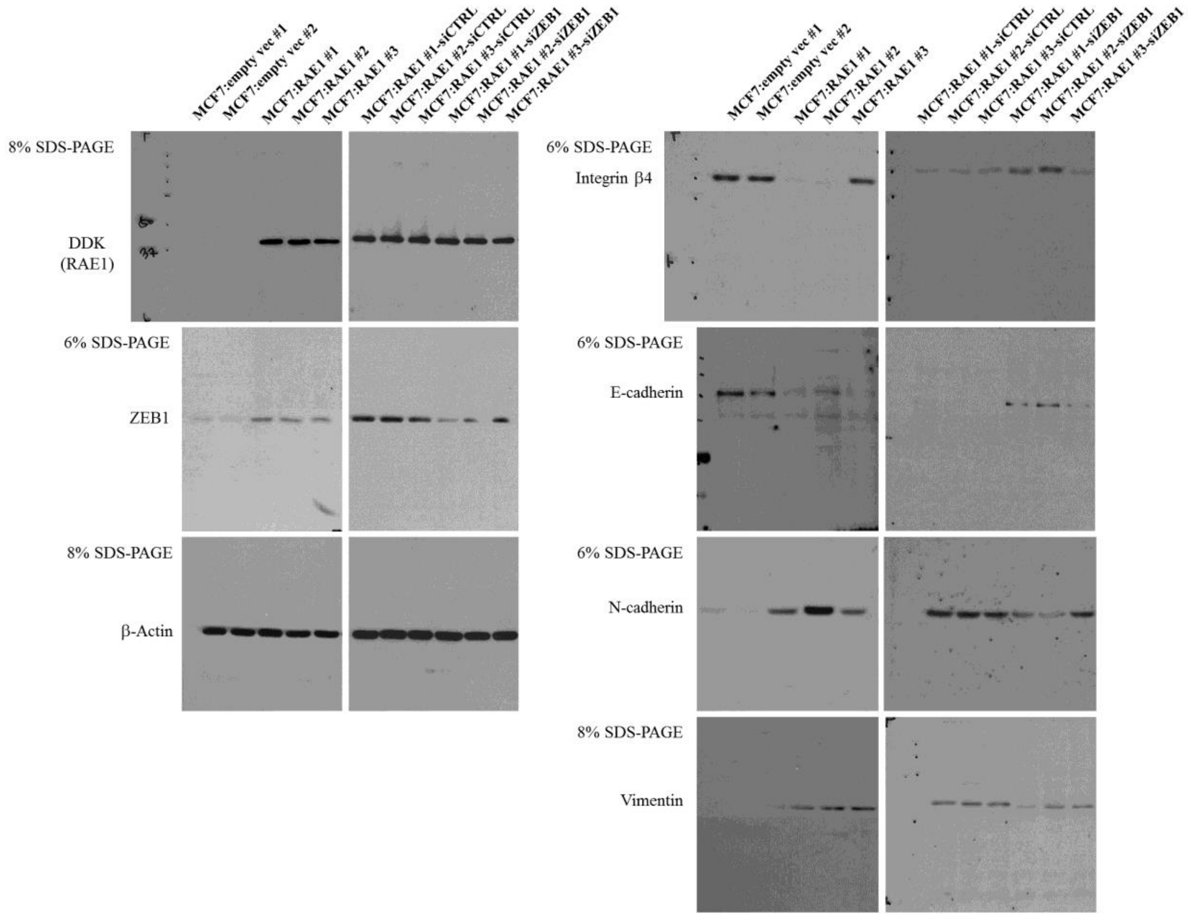
MCF7:empty vec #1
MCF7:empty vec #2
MCF7:RAE1 #1
MCF7:RAE1 #2
MCF7:RAE1 #3
MCF7:RAE1 #1-siCTRL
MCF7:RAE1 #2-siCTRL
MCF7:RAE1 #3-siCTRL
MCF7:RAE1 #1-siZEB1
MCF7:RAE1 #2-siZEB1
MCF7:RAE1 #3-siZEB1
8% SDS-PAGE
DDK
(RAE1)
6% SDS-PAGE
ZEB1
8% SDS-PAGE
β-Actin
MCF7:empty vec #1
MCF7:empty vec #2
MCF7:RAE1 #1
MCF7:RAE1 #2
MCF7:RAE1 #3
MCF7:RAE1 #1-siCTRL
MCF7:RAE1 #2-siCTRL
MCF7:RAE1 #3-siCTRL
MCF7:RAE1 #1-siZEB1
MCF7:RAE1 #2-siZEB1
MCF7:RAE1 #3-siZEB1
6% SDS-PAGE
Integrin β4
6% SDS-PAGE
E-cadherin
6% SDS-PAGE
N-cadherin
8% SDS-PAGE
Vimentin

Figure S4.

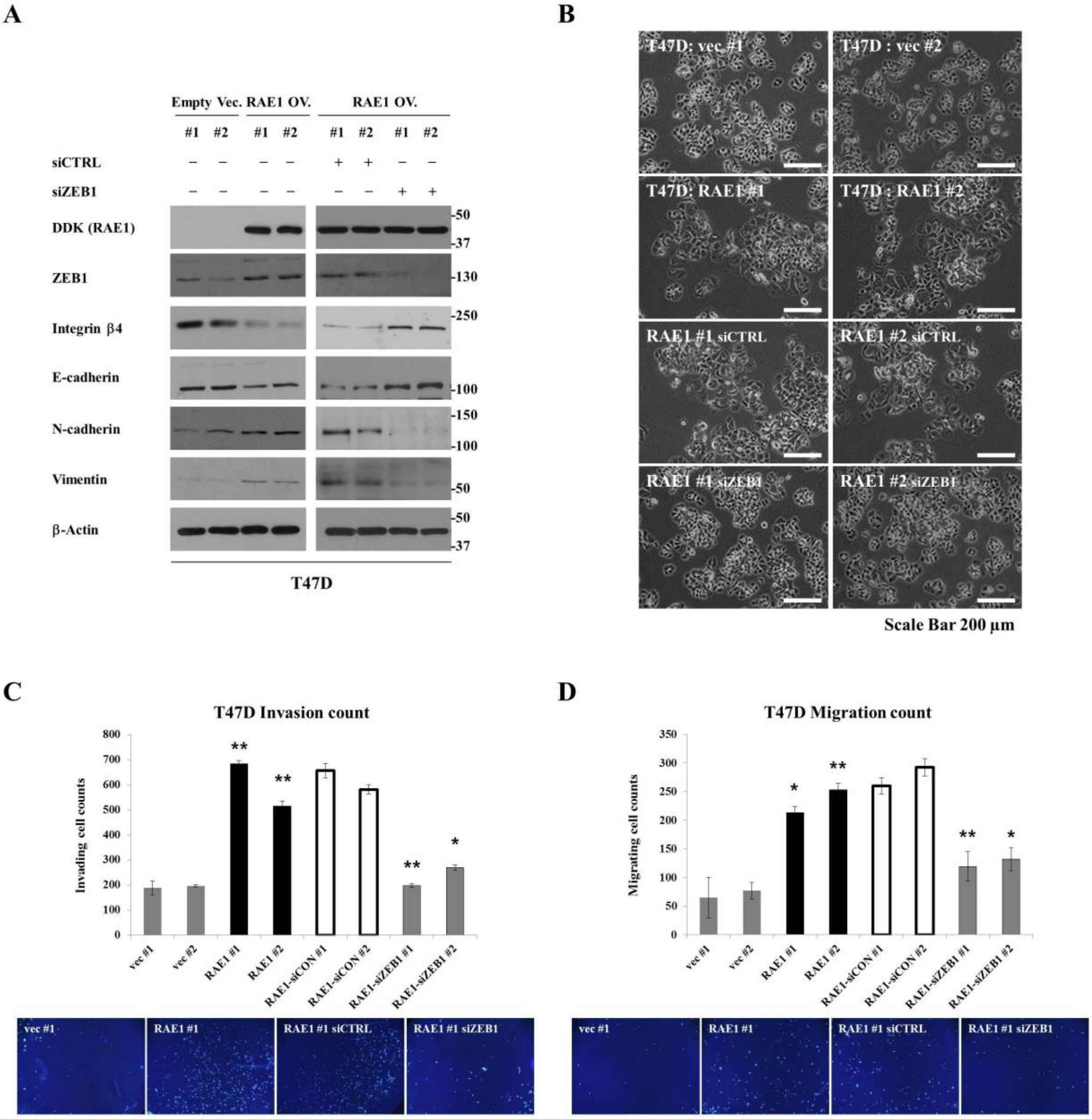
A
Empty Vec. RAE1 OV.
RAE1 OV.
siCTRL
siZEB1
DDK (RAE1)
ZEB1
Integrin β4
E-cadherin
N-cadherin
Vimentin
β-Actin
T47D
B
T47D: vec #1
T47D : vec #2
T47D: RAE1 #1
T47D : RAE1 #2
RAE1 #1 siCTRL
RAE1 #2 siCTRL
RAE1 #1 siZEB1
RAE1 #2 siZEB1
Scale Bar 200 μm
C
T47D Invasion count
Invading cell counts
D
T47D Migration count
Migrating cell counts

Figure S5.

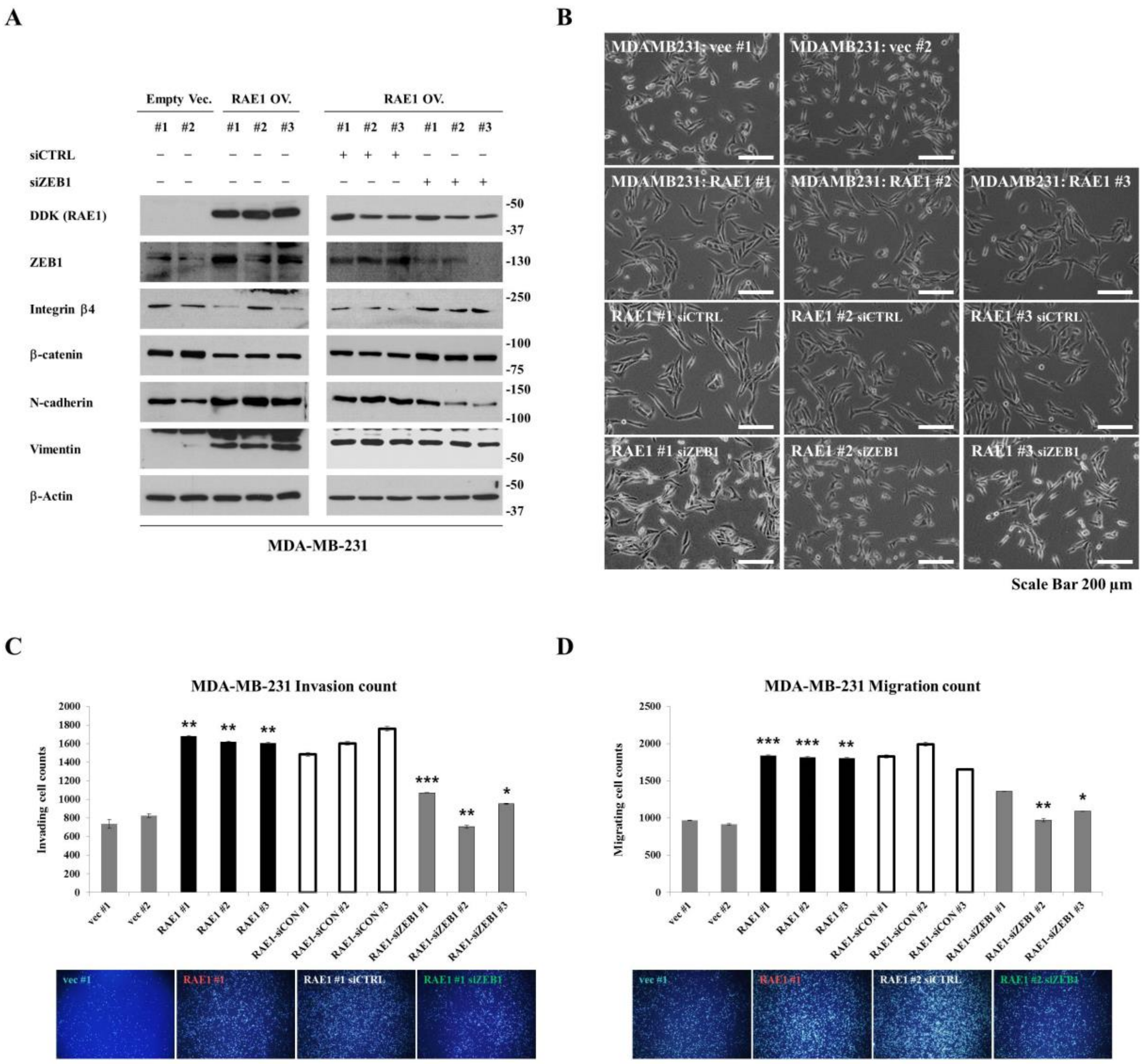

Figure S6.

A

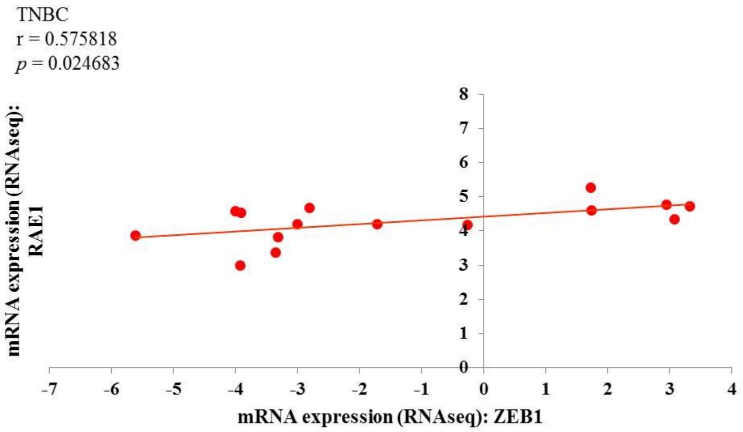
TNBC
r = 0.575818
p = 0.024683
mRNA expression (RNAseq): RAE1
mRNA expression (RNAseq): ZEB1

B

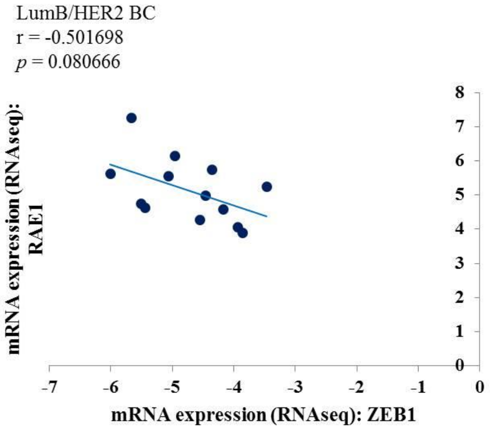
LumB/HER2 BC
r = -0.501698
p = 0.080666
mRNA expression (RNAseq): RAE1
mRNA expression (RNAseq): ZEB1

Figure S7.

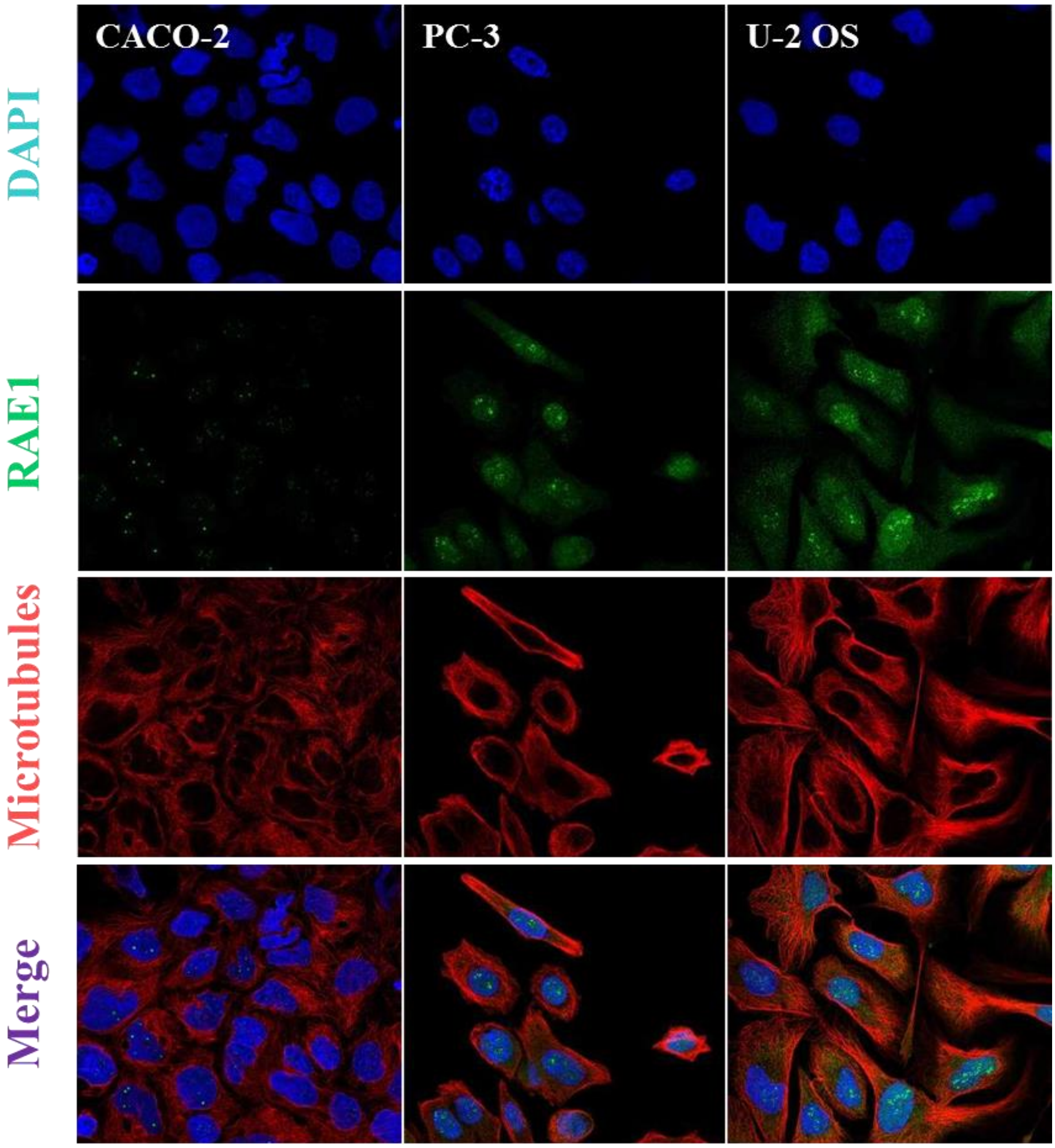

Figure S8.

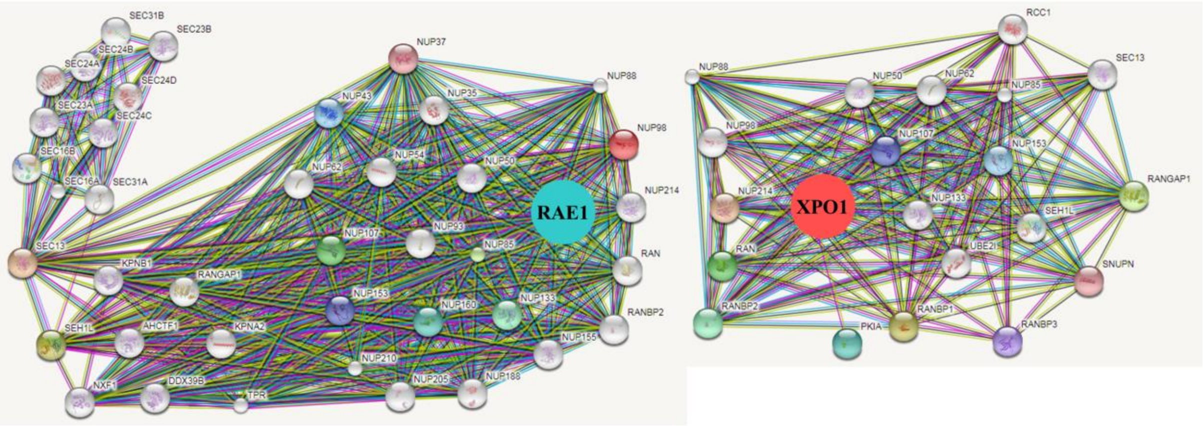
SEC31B
SEC23B
SEC24B
SEC24A
SEC24D
SEC23A
SEC24C
SEC16B
SEC16A
SEC31A
SEC13
NUP37
NUP88
NUP43
NUP35
NUP98
NUP62
NUP54
NUP50
NUP214
RAE1
NUP107
NUP93
NUP85
RAN
KPNB1
RANGAP1
NUP153
NUP160
NUP133
RANBP2
SEH1L
AHCTF1
KPNA2
NUP155
NUP210
NXF1
DDX39B
TPR
NUP205
NUP188
RCC1
NUP88
NUP50
NUP62
SEC13
NUP85
NUP98
NUP107
NUP153
RANGAP1
NUP214
XPO1
NUP133
SEH1L
RAN
UBE2I
SNUPN
RANBP2
RANBP1
PKIA
RANBP3

Figure S9.
